# Synthesis of Water-Soluble Sulfonated Chitin Derivatives for Potential Antioxidant and Antifungal Activity

**DOI:** 10.3390/md20110668

**Published:** 2022-10-26

**Authors:** Fang Luan, Zhenhua Xu, Kai Wang, Xin Qi, Zhanyong Guo

**Affiliations:** 1 Naval Architecture and Port Engineering College, Shandong Jiaotong University, Weihai 264200, China; 2 Key Laboratory of Coastal Biology and Bioresource Utilization, Yantai Institute of Coastal Zone Research, Chinese Academy of Sciences, Yantai 264003, China; 3 University of Chinese Academy of Sciences, Beijing 100049, China

**Keywords:** water-soluble chitin derivative, sulfonated chitin, antioxidant ability, antifungal activity

## Abstract

Chitin is a natural renewable and useful biopolymer limited by its insolubility; chemical derivatization can enhance the solubility and bioactivity of chitin. The purpose of this study was to synthesize novel water-soluble chitin derivatives, sulfo-chitin (SCT) and sulfopropyl-chitin (SPCT), as antioxidant and antifungal agents. The target derivatives were characterized by means of elemental analysis, FTIR, ^13^C NMR, TGA and XRD. Furthermore, the antioxidant activity of the chitin derivatives was estimated by free radical scavenging ability (against DPPH-radical, hydroxyl-radical and superoxide-radical) and ferric reducing power. In addition, inhibitory effects against four fungi were also tested. The findings show that antioxidant abilities and antifungal properties were in order of SPCT > SCT > CT. On the basis of the results obtained, we confirmed that the introduction of sulfonated groups on the CT backbone would help improve the antioxidant and antifungal activity of CT. Moreover, its efficacy as an antioxidant and antifungal agent increased as the chain length of the substituents increased. This derivatization strategy might provide a feasible way to broaden the utilization of chitin. It is of great significance to minimize waste and realize the high-value utilization of aquatic product wastes.

## 1. Introduction

Chitin, comprising β-(1-4)-linked N-acetyglucopyranose units, was first characterized and described in 1884 [1]. It is a linear polysaccharide widely distributed in crustacean shells (such as shrimp, crab and lobster) and the cell walls of fungi, and it is the second most abundant biopolymer on earth. Unlike other forms of biopolymer such as cellulose, chitin has an acetamido group (NHCOCH_3_). Nitrogen-containing compounds have a huge market, with applications such as drug delivery, cell nano patch and biological membranes, etc. [2,3,4,5]. Annually, it has been estimated that chitin is produced in nature on the order of 100 billion tons, and its main source is by-product generated in the crustacean processing industries. It is estimated that 6–8 million tons of crustacean shell waste are discarded annually in the world, resulting in a huge amount of waste and vast disposal cost [6]. One of the economically as well as ecologically sustainable solutions is the extraction of chitin from crustaceans’ shells, which may be a way of minimizing the waste and producing value-added compounds with noteworthy biological properties that can be applied in multifarious fields. 

As a natural renewable resource, chitin has been widely applied due to its complete biodegradability and excellent biocompatibility in combination with non-toxic properties [7,8,9]. Although it is widely available, the insolubility of chitin in water and even most organic solvents becomes the biggest limitation in its practical application. To increase the solubility of chitin, its chemical modification is required. Yet, water-soluble chitin derivatives are mostly obtained by different methods: deacetylation, alkylation, carboxylation and so on [10,11,12]. Among them, sulfonation is an efficient method to improve the water-solubility of derivatives, and polysaccharides exhibit biological activities after sulfation [13,14].

Oxygen in cells can generate reactive oxygen species (ROS) during metabolism, such as hydroxyl radical (•OH), superoxide anion (O_2_^−^), ozone (O_3_), hydrogen peroxide (H_2_O_2_), etc. [15]. Cellular damage by free radicals gives rise to various disorders, for instance chronic renal failure, arthritis, diabetes, sepsis, respiratory distress syndrome, Alzheimer’s disease and cancer [16,17]. Antioxidants have been found to be effective in scavenging these free radicals to protecting cells from various diseases. However, the use of synthetic small-molecule antioxidants has been correlated with detrimental effects to human health and food safety, resulting in rigorous supervision by many governmental agencies [18,19]. With the changes in consumer preferences for safe food, investigations on natural polysaccharides and their unique antioxidant properties have sparked people’s interest. The biocompatibility and biodegradability of chitin and its derivatives, coupled with their capacity to eliminate free radicals, makes them potential functional constituents used in different fields, ranging from food formulations to functional materials and medicine to agriculture [20,21].

Chemical pesticides are usually used to prevent plant pathogens; with the development of the environmentally friendly society, the problems associated with farm chemicals have aroused widespread concern. In recent years, many effective antibacterials have been prohibited due to dangers towards the environment and the health of humans. In this view, it is urgently critical to find new chemical fungistats that not only can hold back the growth of the microorganism effectively, but also are biocompatible, biodegradable and low toxicity. Therefore, novel polysaccharide derivatives emerge as a new class of environmentally friendly biomaterials. 

To the best of our knowledge, there are few papers about chitin derivatives with sulfonated side chains. In the current study, sulfonated chitin could be prepared by modifying chitin using sulfur trioxide pyridine and 1,3-propane sultone, thus obtaining water-soluble derivatives with biological activities. The chitin derivatives designed in this way were expected to have advantageous characteristics such as good antioxidant and antifungal activity. Then, we estimated their antioxidant ability and antifungal activity in vitro, and the relationship between the structure and the antioxidant and antifungal activities of chitin was discussed.

## 2. Results and Discussion

### 2.1. Synthesis and Characterization of Chitin Derivatives 

#### 2.1.1. Synthesis of Chitin Derivatives

**Scheme 1:**
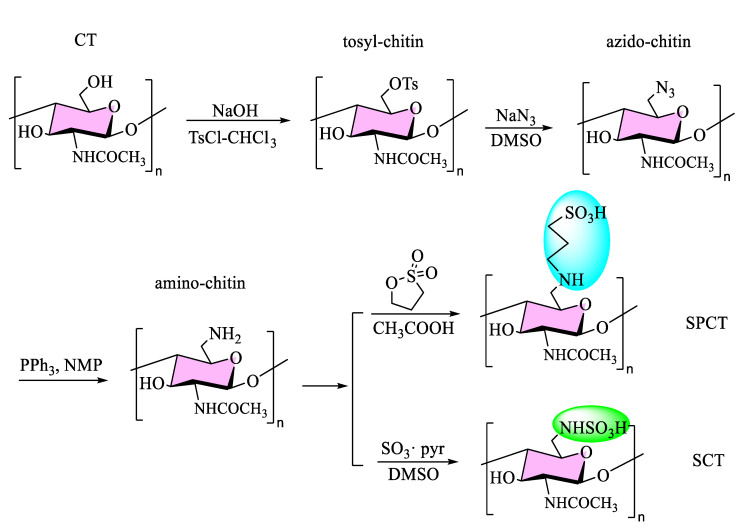
Synthetic route for sulfo-chitin (SCT) and sulfopropyl-chitin (SPCT).

Sulfo-chitin (SCT) and sulfopropyl-chitin (SPCT) were prepared in four steps (Scheme 1), namely tosylation, azidation, reduction, and sulfonation. The first step was to obtain 6-tosyl-chitin through processing chitin (treated with 40% concentrated sodium hydroxide solution) with 4-toluene sulfonyl chloride and chloroform. Afterwards, the azide treatment was used to remove the tosyl group for producing azido-chitin (ACT). Then, amino-chitin (NCT) was successfully synthesized through the reduction reaction of PPh_3_. Raw chitin and all intermediate products (TCT, ACT, NCT) were water insoluble. Lastly, the water-soluble chitin derivatives SCT and SPCT were synthesized by sulfonation. Their solubilities in water were up to 2.0 mg/mL and 6.0 mg/mL, and good water solubility leads to wider use. The enhanced water solubility of derivatives due to the introduction of the (propane) sulfonated group led to an increase in the intermolecular spaces between the chains and partially broke the initial hydrogen bonds. 

The elemental analyses of chitin derivatives and the degree of substitution (DS) are reported in Table 1, as calculated by the S/N (TCT, SCT and SPCT) or C/N (other derivatives) ratio obtained from the elemental analysis. 

#### 2.1.2. FTIR Analysis

**Figure 1:**
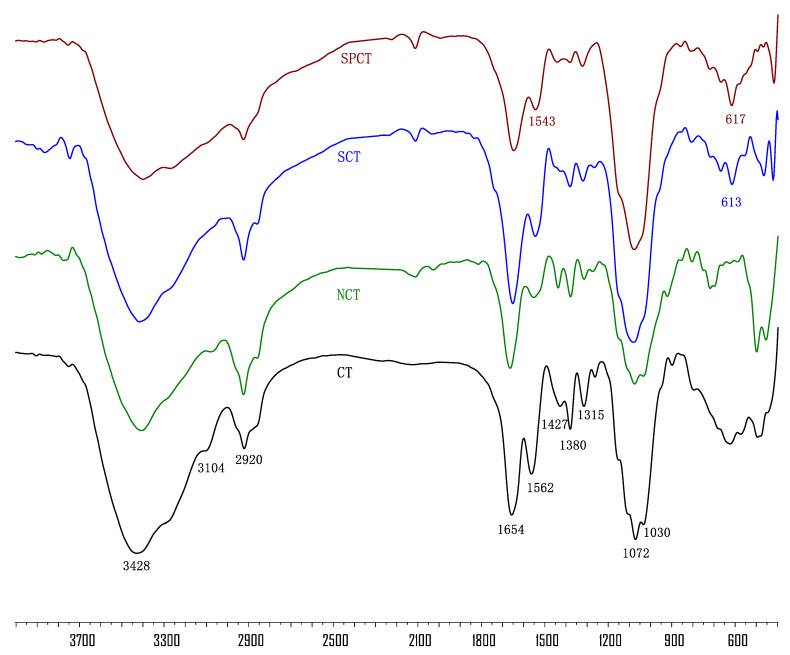
IR spectra of chitin and its derivatives.

The formation of chitin and its derivatives were also confirmed via FTIR spectroscopy (Figure 1).

The spectrum of unmodified chitin (CT) indicates that the polysaccharide mainly contains the following characteristic bands: 3444 and 3104 cm^−1^ (–OH and –NH), 2922 cm^−1^ (aliphatic –CH), 1662, 1562 and 1315 cm^−1^ (amide I, II and III), 1427 cm^−1^ (pyranose ring), 1377 cm^−1^ (acetamide groups) and 1072 and 1030 cm^−1^ (C–O) [22].

In the NCT spectra, the bending vibration bands of –NH_2_ groups at around 1600 cm^−1^ was not observed, which may be attributable to the influence of amide. Moreover, the absorption intensities at 1030 cm^−1^ decrease, indicating successful incorporation of the amine group.

For SCT, the new peak at 613 cm^−1^, attributed to the S=O stretching vibration of the sulfonic acid group [23,24], suggests that a sulfonation reaction took place on the amine group and the synthesis of the desired compound was achieved.

Finally, for SPCT, the new peaks at 617 cm^−1^, corresponding to the stretching vibration of the SO_2_ group in sulfonic acid, suggest the presence of the SO_3_H group in SPCT; the peak at 1543 cm^−1^ was assigned to the C–N–C bending vibration of the SPCT branch, suggesting that reaction occurred at the amine group to produce sulfopropyl-chitin [13]. 

#### 2.1.3. NMR Analysis

**Figure 2:**
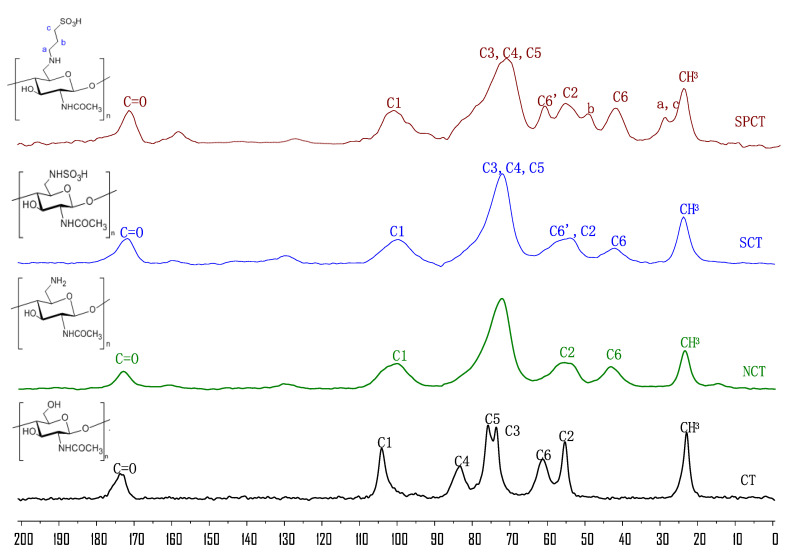
Solid-state ^13^C NMR spectra of chitin and its derivatives.

Solid-state ^13^C NMR spectroscopy was applied to further elucidate the chemical structures of chitin and its derivatives (Figure 2).

In the ^13^C NMR spectrum of chitin (CT) shows eight peaks: one at 104 ppm for C1, the second at 83 ppm for C4, the third at 76 ppm for C5, the fourth at 74 ppm for C3, the fifth at 61 ppm for C6 and the sixth at 55 ppm for C2. The other signals at 173 and 23 ppm are assigned to C=O and CH3.

In the spectra of NCT, peaks ranging from 85 ppm to 64 ppm were assigned to C3, C4 and C5. At the same time, the peak at 61 ppm shifted to a higher field (43 ppm) compared with the signals of C-6 of CT. This indicates that the amino groups were introduced into chitin successfully.

In the SCT spectrum, the C6-NH_2_ participation in the reaction was marked as C6’ and that which was not involved was marked as C6. The C6’ signal shifted from 42 to 57 ppm. In addition, the signal at 42 ppm was still observed, which adequately indicates that the C6-NH_2_ moiety was only partially replaced in SCT.

Similarly, in the SPCT spectra, the C6-NH_2_ participation in the reaction was marked as C6′ and C6-NH_2_, and that which was not involved was marked as C6. The signal at 61 ppm was attributed to the substituted C6′; furthermore, the signal at 42 ppm was assigned to partial replacement at C6. This implies that free amino groups at C6 were incompletely replaced. Two new signals at 27 ppm and 48 ppm appeared, which were assigned to the carbons in 1,3-propane sultone [25]. According to the results, we speculate that the amino groups reacted with the 1,3-propane sultone and the successful synthesis of the desired chitin derivatives was achieved.

#### 2.1.4. Thermogravimetric and Derivative Thermogravimetric Analysis (TGA/DTG)

**Figure 3:**
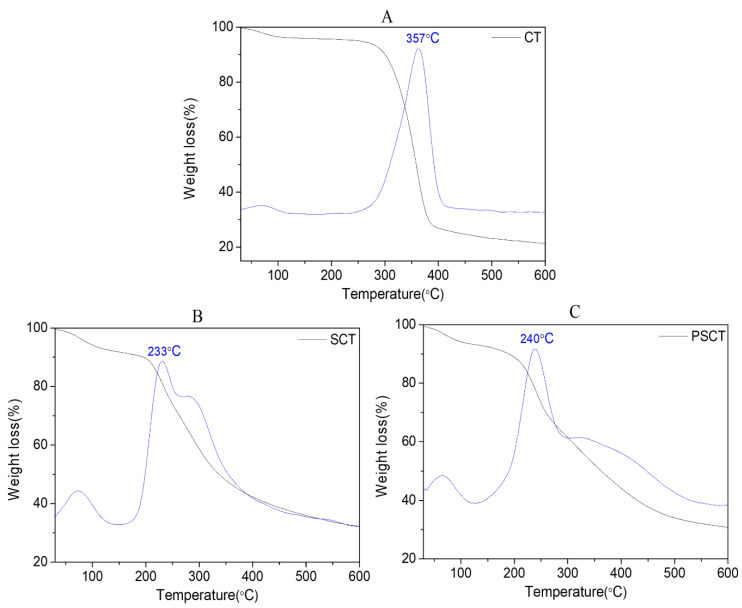
TGA thermogram and DTG curves. (**A**) CT, (**B**) SCT, (**C**) SPCT.

The thermogravimetric analyses (TGA) and the corresponding derivative thermogravimetric (DTG) curves of CT, SCT and SPCT are represented in Figure 3. All the TGA curves of samples exhibit two stages of weight loss. CT underwent a 4.4% loss of mass from 45 °C to 140 °C, which was attributed to evaporation of water already within the polymer structure [26]. In the following step, a sudden decrease in weight was observed at about 260–396 °C (DTGmax at 357 °C); 72.2% mass loss was observed. This mass loss could be ascribed to the decomposition of the saccharide structure of the chitin molecule, which contains the dehydration of saccharide rings and the disintegration of both the N-acetylated units as well as the degradation of other proteins associated with the chitin structure [27,28]. 

Similar TGA behavior was seen for SCT and SPCT. Firstly, the TGA curve of SCT exhibited a 7.4% loss of mass from 45 °C to 160 °C. Then, the major weight loss of SCT (59.8%) occurred at about 200–600 °C (DTGmax at 233 °C). The following loss was likely caused by the declined hydrogen bonding and disruption of glycoside linkages. In the case of SPCT, a comparatively similar behavior was observed as seen in SCT. The TGA curve of SPCT exhibited a 7.9% loss of mass from 40 °C to 152 °C, followed by a 61.2% loss of mass at about 160–600 °C (DTGmax at 240 °C). In addition, the initial decomposition temperatures of CT, SCT and SPCT were found to be 260, 200 and 160 °C, respectively. Compared to CT, SCT and SPCT have lower initial decomposition temperatures, and SCT and SPCT are thermally instable due to the introduction of the (propane) sulfonated group, which disrupted the H-bond formation between the chains, as discussed in the solubility test.

Comprehensively, it is reasonable to presume that chitin derivatives were more susceptible to the thermal decomposition temperature than raw chitin. A possible reason is that chemical reactions lowered the regularity of chitin molecular chain and resulted in the breakage of hydrogen bonds, thereby reducing its resistance to thermal degradation. Our results were in accordance with previous studies [29,30].

#### 2.1.5. X-ray Diffraction (XRD) Analysis

**Figure 4:**
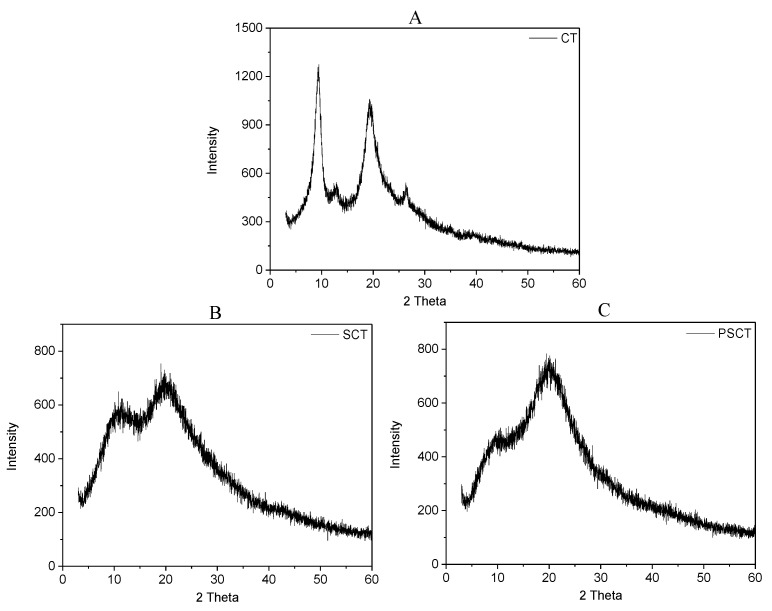
XRD spectra of CT (**A**), SCT (**B**) and SPCT (**C**).

The crystalline structure of chitin and its derivatives were analyzed via X-ray diffraction (Figure 4). CT exhibited four peaks at 9.4°, 12.8°, 19.5° and 25.2°. Two of them are faint (12.8° and 25.2°) and the other two are sharp (9.4° and 19.5°). Compared to CT, the XRD patterns of SCT and SPCT exhibited some changes in their peak width, peak intensity and angles. The sharp peak of CT at 9.4° moved to 10.5° and 10.8° in the patterns of SCT and SPCT, owing to the introduction of sulfonated groups and propane sulfonated groups to the structure of chitin. In addition, for SCT, the peak at 19.5° shifted to 19.9°, its intensity decreased and its width increased. Similarly, for SPCT, the peak at 19.5° shifted to 20.6°, and the intensity and width of the peak had the same changes as well. Meanwhile, the diffraction peak at 2θ = 12.8° and 25.2° disappeared, which may be due to the damaging of inter- and intra-molecular hydrogen bonds [31]. The results demonstrate that the (propane) sulfonated group grafted on chitin resulted in a decrease in crystallinity, which can be observed in the form of relatively weaker reflection in the spectra of SCT, and SPCT. A lower crystallinity index leads to better water solubility [32]; as a result, SCT, and SPCT should have a much better aqueous solubility than chitin, which is consistent with the solubility test.

### 2.2. Antioxidant Activities

**Figure 5:**
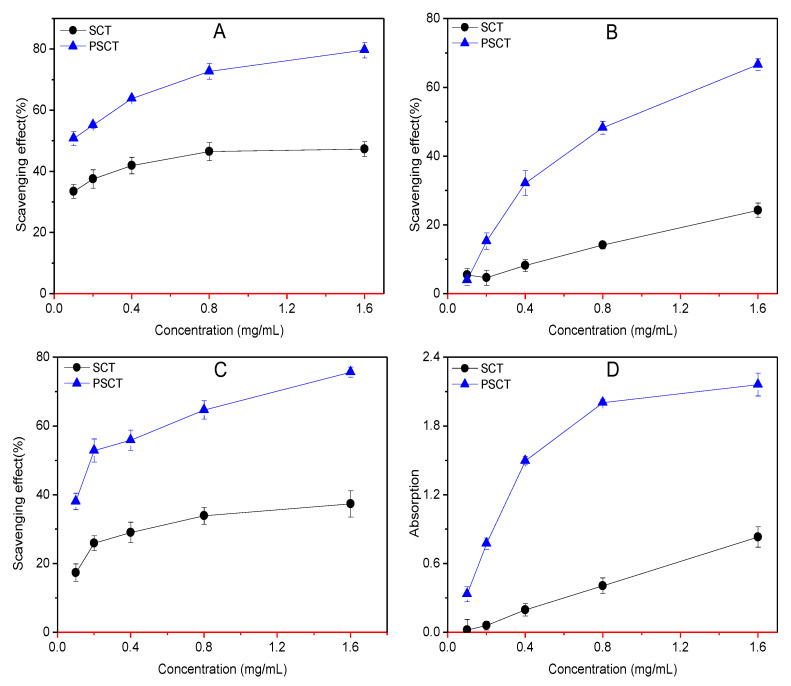
Antioxidant effect of samples: (**A**) DPPH-radical scavenging ability; (**B**) hydroxyl-radical scavenging ability; (**C**) superoxide-radical scavenging ability; (**D**) reducing power.

The antioxidant assay of chitin could not be performed owing to its insolubility. Therefore, the default is 0 (red line in Figure 5).

#### 2.2.1. DPPH-Radical Scavenging Ability Assay

The DPPH-radical scavenging activity of SCT and SPCT is illustrated in Figure 5A. In this experiment, we can clearly see that the scavenging effect increased with the increasing concentration. Meanwhile, the scavenging capability against DPPH-radical was in order of SPCT > SCT > CT at the concentrations studied. Moreover, SPCT and SCT show antioxidant activities at 1.6 mg/mL of 79.7% and 47.3%, respectively. 

The scavenging activity may be correlated with DPPH-radicals reacting with active hydrogen in chitin derivatives to form a stable macromolecule. The more active the hydrogen, the higher the scavenging capability. Therefore, the scavenging effect of all samples increased with increasing concentration. The sulfonated groups grafted on CT are able to act as hydrogen donors. Furthermore, SPCT is more reactive than SCT as it is sterically less hindered; as a result, the hydrogens of the derivative are more exposed to the outside. 

Meanwhile, the 50% inhibition concentration (IC_50_) is a good parameter to evaluate scavenging capability. A lower IC_50_ value indicates a greater antioxidant activity. According to the results, SPCT had the highest activity compared to CT and SCT. The IC_50_ of SPCT was 0.10 mg/mL. In other words, the SPCT can act as a reducing agent, and propane sulfonated groups play an important role in scavenging activity against DPPH-radicals. 

#### 2.2.2. Hydroxyl-Radical Scavenging Ability Assay

There is no doubt that timely removal of excess hydroxyl radicals is crucial to create a healthier body. From Figure 5B, we can clearly see that the scavenging capability against hydroxyl-radicals was in the order of SPCT > SCT > CT at the concentrations studied. Results indicate that SPCT and SCT exhibited enhancement on hydroxyl-radical scavenging capability; the scavenging effects at 1.6 mg/mL were 66.6% and 24.3%, respectively. In additional, IC_50_ of SPCT was 0.86 mg/mL.

The scavenging activity may be correlated with active hydrogen in chitin derivatives. Active hydrogen in the polysaccharide unit can react with ·OH by the typical H abstraction reaction. The grafting of propane sulfonated groups onto CT can donate more protons to free radicals; this principle was discussed in the DPPH-radical assay. These results suggest that sulfonated groups could be an important factor in affecting hydroxyl-radical scavenging activity.

#### 2.2.3. Superoxide-Radical Scavenging Ability Assay

Superoxide scavenging activity was determined in the NBT assay. According to Figure 5C, the result concurred with the scavenging properties against DPPH-radicals. Moreover, the scavenging capability of the samples increased with the increase in concentration at 0.1–1.6 mg/mL. The maximum of 37.4% inhibition was observed at the concentration of 1.6 mg/mL of SCT. SPCT also had the strongest scavenging activity, reaching 75.7% at 1.6 mg/mL. Moreover, the IC_50_ of SPCT was 0.17 mg/mL.

As reported, the scavenging effect is correlated with the number of active hydrogens in the molecule. As mentioned in the DPPH-radical and hydroxyl-radical assay, SCT and SPCT can be proton donors that react with superoxide anions. The results clearly indicate that SPCT can be regarded as an efficient antioxidant polymer and propane sulfonated groups undoubtedly play crucial roles in its free radical scavenging capability.

#### 2.2.4. Reducing Power Assay

The reducing powers of chitin and its derivatives are shown in Figure 5D. The results clearly indicate that the reducing power of samples exhibited an upward trend with the increase in sample concentration. SPCT exhibited stronger reducing power than SCT, and they were found to have reducing powers at 1.6 mg/mL of 2.2 and 0.8, respectively. 

Accordingly, the reducing capacity of a compound may serve as a significant indicator of its potential antioxidant activity [33]. Based on the results, it is concluded that propane sulfonated groups can enhance the ability of the reducing power.

These data demonstrate that the scavenging effect of all samples increased with increasing concentration. Additionally, the scavenging abilities were in order of SPCT > SCT > CT in the tested concentration. It is valid to find that the antioxidant ability increases with the increasing chain length of alkyl substituent. This observation is in agreement with previous reports [34]. According to the results, we could conclude SPCT with presumed antioxidant properties may be developed into new antioxidants as a possible food ingredient or supplement in the pharmaceutical industry.

### 2.3. Antifungal Activity

**Figure 6:**
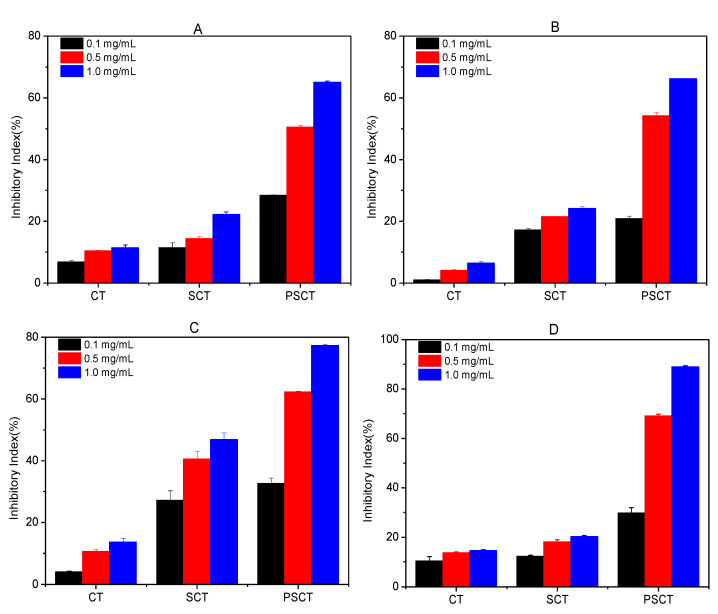
Antifungal activity against *P. asparagi* (**A**), *F. oxyspirum* f. *niveum* (**B**), *B. cinerea* (**C**), and *F. oxysporum* f. sp. *Cucumerium* (**D**) at 0.1, 0.5 and 1.0 mg/mL.

The action of phytopathogenic fungi causes severe damage to plants; thus, control of these plant-threatening fungi is of great interest. Here, we tested CT, SCT and SPCT against destructive plant-threatening fungi (*P. asparagi*, *F. oxyspirum* f. *niveum*, *B. cinerea* and *F. oxysporum* f. sp. *Cucumerium*). The results are shown in Figure 6. It is clear from the plots that the antifungal activities of the derivatives correlated well with the increase in concentration. Additionally, chitin derivatives exhibited the best antifungal activity at 1.0 mg/mL. Simultaneously, the inhibitory index was in the order of SPCT > SCT > CT. Details are as follows.

As depicted in Figure 6A, CT and SCT slightly inhibited the growth of *P. asparagi* and the inhibitory rates were 11.5% and 22.3% at 1.0 mg/mL, respectively. Compared to both, SPCT had better antifungal activity, and the inhibitory index was up to 65.1% at 1.0 mg/mL. In other words, SPCT possesses powerful antifungal activity against *P. asparagi,* and propane sulfonated groups are an important factor that affects antifungal activity.

As depicted in Figure 6B, CT also slightly inhibited the growth of *F. oxyspirum* f. *niveum* and the inhibitory rate was 6.5% at 1.0 mg/mL. Compared with CT, the inhibitory indices of SCT and SPCT at 1.0 mg/mL were 24.2% and 66.2%, respectively. Namely, both SCT and SPCT have a better ability to inhibit *F. oxysporum*, and propane sulfonated groups might help to improve the antifungal activity of chitin derivatives.

The antifungal assays against *B. cinerea* suggest that the antifungal activities of the chitin derivatives exhibited a dose-dependent manner (Figure 6C). It is evident that the antifungal activity of the chitin derivatives was much better than that of chitin at the same concentration. The inhibitory rates of CT, SCT and SPCT against *B. cinerea* at 1.0 mg/mL were 13.6%, 46.9% and 77.3%, respectively. 

The inhibitory rates of CT, SCT and SPCT against *F. oxysporum* f. sp. *Cucumerium* are shown in Figure 6D. The results are similar to the antifungal activity against *P. asparagi*. The inhibitory rate of CT was 14.6% at 1.0 mg/mL, while those of SCT and SPCT at 1.0 mg/mL were 20.3% and 89.0%, respectively. 

Based on the results mentioned above, sulfonated chitin can serve to repress the growth of fungus, and SPCT with propane sulfonated groups exhibits higher antifungal activity than SCT with sulfonated groups and CT. The result also concurs with the previous studies reported by Sajomsang and Li, in which they also discovered that the antifungal activity of chitosan derivatives against the plant pathogenic fungi was enhanced by the increasing chain length of alkyl substituent [35,36]. According to the results mentioned above, propane sulfonated groups are considered efficacious antifungal groups. Therefore, SPCT that exhibits selective bactericidal activity could be used in agricultural industries.

## 3. Materials and Methods

### 3.1. Materials

Chitin was purchased from Sinopharm Chemical Reagent Co., and its degree of deacetylation was 10%. The other reagents, such as chloroform, sodium azide, triphenylphosphine (Ph_3_P), 4-toluene sulfonyl chloride, 1,3-propanesulfonate, sulfur trioxide pyridine, N-Methyl pyrrolidone (NMP) and dimethyl sulfoxide (DMSO), etc., were provided by Sinopharm Chemical Reagent Co., Ltd., Shanghai, China.

### 3.2. Analytical Methods

FT-IR spectra of the samples diluted in KBr pellets were performed on a Fourier transform infrared spectrometer (JASCO Co., Ltd. Shanghai, China). The elemental analyses (C, H, and N) were carried out using a Vario Micro Elemental Analyzer (Elementar, Germany). The UV–Vis absorbance was measured using a T6 New Century UV spectrometer (P General Co., Ltd., Beijing, China). ^13^C nuclear magnetic resonance (^13^C NMR) spectra were carried out on a Bruker AVANCE III spectrometer (Bruker Tech. and Serv. Co., Ltd. Beijing, China.) The thermogravimetric analysis (TGA) was recorded on the TGA/DSC1/1100 (Mettler-Toledo). The X-ray patterns of samples were measured using an X-ray diffractometer (D8 advance, Bruker, Germany).

### 3.3. Synthesis

As shown in Scheme 1, amino-chitin was synthesized according to the earlier literature [37]. The synthesis and characterization of amino-chitin are provided in the Supplementary Materials. SCT and SPCT were prepared as follows.

#### 3.3.1. Preparation of Sulfo-Chitin (SCT)

A mixture of 0.3 g amino-chitin and 80 mL DMSO was treated with sulfur trioxide pyridine complex (1.2 g) at 80 °C for 24 h. The crude product was dialyzed for 2 days and lyophilized. Yield: 64%.

#### 3.3.2. Preparation of Sulfopropyl-Chitin (SPCT)

We dispersed 0.3 g of amino-chitin into 50 mL of 2% acetic acid solution via magnetic stirring. Then, 1.0 mL of 1,3-propanesulfonate was added into solution, and the solution was raised to 80 °C and maintained for 24 h. The crude product was dialyzed for 2 days and lyophilized. Yield: 40%.

### 3.4. Investigation of the Antioxidant Activity

Antioxidant activity models can be classified into two basic mechanisms: single electron transfer (SET) and hydrogen atom transfer (HAT). The SET mechanism studies one electron’s transferring ability to reduce metals and radicals with changes in color as a result, such as 2,2-diphenyl-1-picrylhydrazyl radical (DPPH) scavenging, hydrogen peroxide scavenging and ferric ion reducing antioxidant power. On the contrary, the HAT mechanism is the ability to quench free radicals by hydrogen donation, which can be measured by various assays such as oxygen radical absorbance and total oxyradical scavenging capacity assay.

#### 3.4.1. DPPH-Radical Scavenging Activity

The DPPH scavenging activities of the samples were determined using the method described by Luan [38]. Testing samples (SCT and SPCT) and 180 μM DPPH ethanol solution were incubated for 0.5 h at 25 °C. Afterwards, the reaction system was shaken evenly and incubated in dark for 20 min. Finally, the absorbance of the mixture was recorded at 517 nm spectrophotometrically. The assay was performed in triplicate and the DPPH-radical scavenging activity was computed using the following Equation (1):

(1) $$\mathrm{Scavenging}\;\mathrm{effect}\;\left(\%\right)=\left[1-\frac{A_{\mathrm{sample}\;517\;\mathrm{nm}}-A_{\mathrm{control}\;517\;\mathrm{nm}}\;}{A_{\mathrm{blank}\;517\;\mathrm{nm}}}\right]\times 100$$

where *A*_sample 517 nm_ is the absorbance of the sample at 517 nm; *A*_control 517 nm_ is the absorbance of the control at 517 nm; and *A*_blank 517 nm_ is the absorbance of the blank at 517 nm.

#### 3.4.2. Hydroxyl-Radical Scavenging Activity

The hydroxyl-radical scavenging power of the samples was performed in accordance with the approach of Sun [39]. The reaction mixture, with a total volume of 4.5 mL and involving testing samples (SCT and SPCT), was incubated with EDTA-Fe^2+^ (220 μmol/L), H_2_O_2_ (60 μmol/L) and safranine T (0.23 μmol/L) in phosphate buffer (pH 7.4) for 0.5 h at 37 °C under shaken condition. The absorbance of the resulting solution was recorded at 520 nm against a blank. The assay was performed in triplicate and the hydroxyl-radical scavenging activity was computed using the following Equation (2):

(2) $$\mathrm{Scavenging}\;\mathrm{effect}\;\left(\%\right)=\frac{A_{\mathrm{sample}\;520\;\mathrm{nm}}-A_{\mathrm{blank}\;520\;\mathrm{nm}}\;}{A_{\mathrm{control}\;520\;\mathrm{nm}}-A_{\mathrm{blank}\;520\;\mathrm{nm}}}\times 100$$

where *A*_sample 520 nm_ is the absorbance of the sample at 520 nm; *A*_control 520 nm_ is the absorbance of the control at 520 nm; and *A*_blank 520 nm_ is the absorbance of the blank at 520 nm.

#### 3.4.3. Superoxide-Radical Scavenging Activity

The superoxide-radical scavenging capability was determined based on the procedure reported with slight modifications [40]. It involving testing samples (SCT and SPCT), reduced nicotinamide adenine dinucleotide (338 μmol), phenazine mothosulfate (30 μmol) and nitro blue tetrazolium (72 μmol) in Tris-HCl buffer (16 mM, pH 8.0). After the resulting solution was incubated for 5 min at room temperature, the absorbance was read quickly at 560 nm. The assay was performed in triplicate and the superoxide-radical scavenging effect was computed according to the following Equation (3):

(3) $$\mathrm{Scavenging}\;\mathrm{effect}\;\left(\%\right)=\left[1-\frac{A_{\mathrm{sample}\;560\;\mathrm{nm}}-A_{\mathrm{control}\;560\;\mathrm{nm}}\;}{A_{\mathrm{blank}\;560\;\mathrm{nm}}}\right]\times 100$$

where *A*_sample 560 nm_ is the absorbance of the sample at 560 nm; *A*_control 560 nm_ is the absorbance of the control at 560 nm; and *A*_blank 560 nm_ is the absorbance of the blank at 560 nm.

#### 3.4.4. Reducing Power Activity

The reducing power was measured following the earlier methods [41]. In summary, 1.5 mL of 1% potassium ferricyanide was mixed with 1.5 mL of testing sample (CT, SCT and SPCT), and the resulting solution was incubated at 50 °C for 20 min. Then, 10% trichloroacetic acid (1.5 mL) was added. Subsequently, the upper layer (2.0 mL) was blended with distilled water (2.0 mL) and 0.1% ferric chloride (0.2 mL). After standing undisturbed for 10 min, the absorbance of the mixture was recorded at 700 nm. The reducing power of the samples increased with the absorbance.

### 3.5. Evaluation of Antifungal Activity In Vitro

Antifungal assays were determined according to the method reported by Zhang [42]. In brief, each sample solution (CT, SCT and SPCT) was added to fungal medium to give a final concentrations of 0.1, 0.5 and 1.0 mg/mL and sterilized by autoclaving at 120 °C for 40 min. After the PDA medium was cooled, the fungal mycelia disk (5 mm) was transferred to the nutrient agar plate and incubated at 27 °C. All the samples were plated in triplicate on agar plates, and the inhibition rate was calculated as follows with Equation (4):

(4) $$\mathrm{Antifungal}\;\mathrm{index}\;\left(\%\right)=\left[1-\frac{D_{a}\;}{D_{b}}\right]\times 100$$

where *D*_a_ is the diameter of growth zone in the test plate and *D*_b_ is the diameter of growth zone in the control plate. 

### 3.6. Statistical Analysis

All data were reported as means ± standard deviation. The differences in the assays were determined via Scheffe’s method. Data were analyzed by the analysis of variance to guarantee statistical significance.

## 4. Conclusions

In summary, chitin derivatives with propane sulfonated groups were successfully synthesized. In addition, the antioxidant and antifungal activities of chitin and sulfonated chitin derivatives were tested in vitro. Chemical derivatization and the incorporation of (propane) sulfonated groups were done on chitin to yield better solubility, antioxidant and antifungal activities. We found that antioxidant abilities and antifungal properties were in the order of SPCT > SCT > CT. From the results, it can be inferred that the antioxidant ability and antifungal activity increased with an increase in the chain length of alkyl substituents. At the same time, propane sulfonated groups led to an enhancement of the antioxidant and antifungal activity. Furthermore, chitin derivatives with enhanced biological activities could be utilized as potential biomaterial for antioxidant and antifungal applications. Therefore, this work can offer a feasible way to overcome chitin’s limitation by generating soluble derivatives. Undoubtedly, the further modification and utilization of chitin can minimize the waste of shell-waste and create new industrial opportunities.

## Figures and Tables

**Table 1:** The elemental analyses and the degrees of substitution of chitin derivatives.

Full Name	Sample (Abbreviations)	Found (%)				DS	Formula
		C	H	N	S		
chitin	CT	45.23	7.19	6.59		1.0	C_8_H_13_NO_5_
tosyl-chitin	TCT	50.02	6.56	4.53	7.86	0.93	(C_15_H_19_NO_7_S)_0.89_(CT)_0.11_
azido-chitin	ACT	44.25	5.22	20.87		0.90	(TCT)_0.1_ (C_8_H_12_N_4_O_4_)_0.9_
amino-chitin	NCT	46.95	6.80	14.97		0.87	(ACT)_0.04_ (C_8_H_14_N_2_O_4_)_0.87_
sulfo-chitin	SCT	36.90	5.85	9.84	8.74	0.40	(NCT)_0.6_(C_8_H_14_N_2_O_7_S)_0.4_
sulfopropyl-chitin	SPCT	39.62	6.11	9.88	8.14	0.41	(NCT)_0.59_(C_11_H_19_N_2_O_7_S)_0.41_

## Supplementary Materials

The following supporting information can be downloaded at: https://www.mdpi.com/article/10.3390/md20110668/s1, Figure S1: FTIR spectra of TCT and ACT; Figure S2: Solid-state ^13^C NMR spectra of TCT and ACT.
