# Supplementary material for: Synthesis of Water-Soluble Sulfonated Chitin Derivatives for Potential Antioxidant and Antifungal Activity
Source: Mar Drugs. 2022 Oct 26;20(11):668. doi: 10.3390/md20110668 (PMC9697452; doi:10.3390/md20110668)
Supplement: Supplementary file 1 [file marinedrugs-20-00668-s001.zip › marinedrugs-1938832-supplementary.pdf]

Synthesis of Water-soluble Sulfonated Chitin Derivatives for Potential Antioxidant and Antifungal Activity

Synthesis of polymeric amino-chitin

1. *Preparation of tosyl-chitin (TCT)*

A mixture of 1.0 g of chitin and 20 mL of 42 % aqueous sodium hydroxide was left standing at a reduced pressure for 2 h. To this was added 50 g of crushed ice, and the mixture was stirred to give a clear solution. It was then cooled in an ice bath, and 7.5 g (7.5 mol equivalent to pyranose rings) of tosyl chloride dissolved in 20 mL of chloroform was added with stirring. After 2 h of reaction, the ice bath was replaced, and the mixture was stirred at room temperature for another 2 h. The mixture was gradually poured into water, and the precipitate was washed with water until neutral. Then the precipitate was washed with ethanol and dried at 60°C. The product was obtained as a white solid.

2. *Preparation of azido-chitin (ACT)*

A mixture of 2.5 g tosylation of chitin and 80 mL DMSO was treated with sodium azide (3.6 g) at 90°C for 6 h. The resulting mixture was cooled to room temperature and then poured into 400 mL ethyl acetate. The precipitate was collected, washed with ethanol, and then dried at 60°C to give the product.

3. *Preparation of amino-chitin (NCT)*

Azidation of chitin (0.63 g) was suspended in 65 mL of DMF and 2.83 g of triphenylphosphine was added. After 24 h of reaction at room temperature, 1.3 mL water was then added to the mixture. After another 24 h of reaction, the resulting solid was washed with 200 mL of ethanol, and dried to give the product.

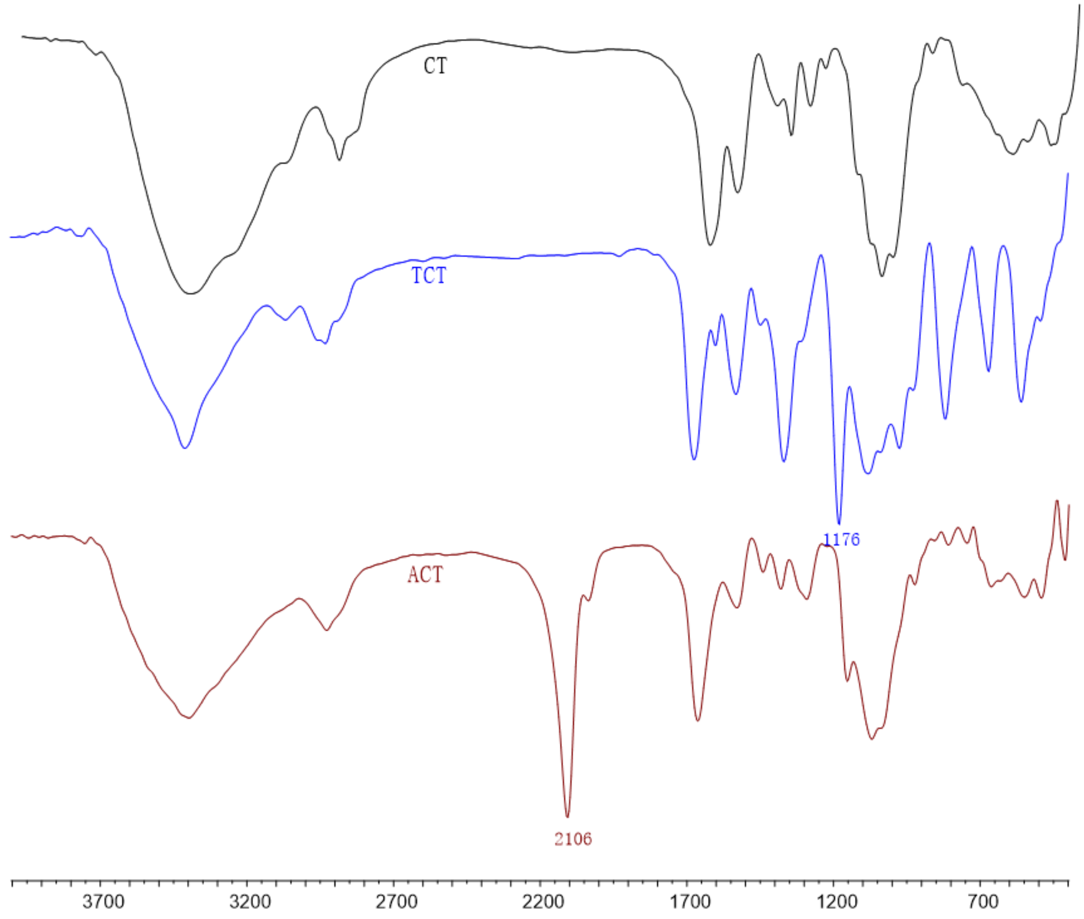

Figure S1. FTIR spectra of TCT and ACT.

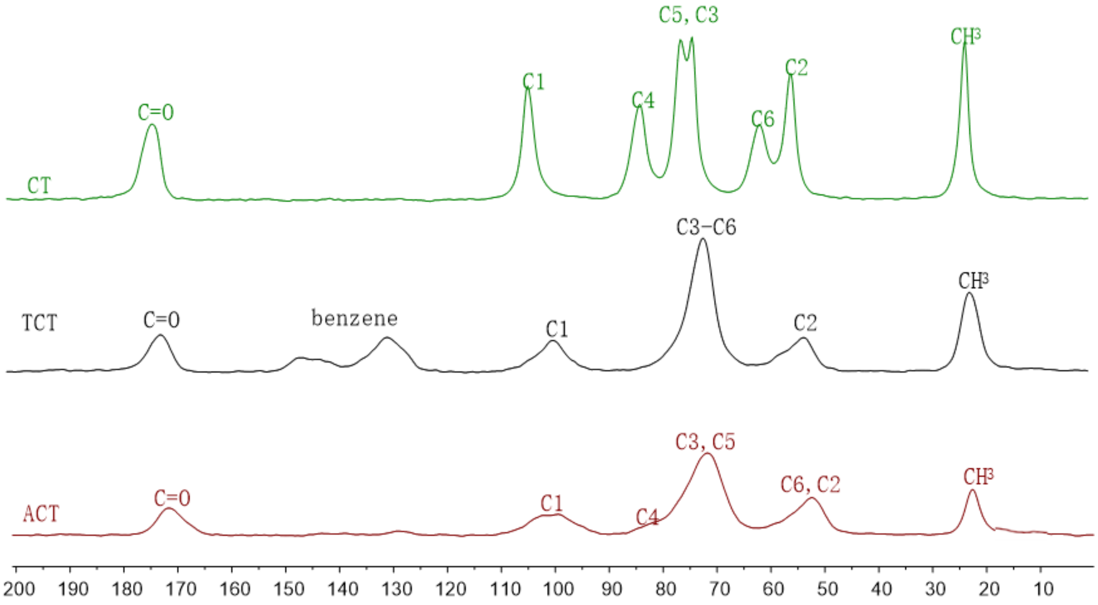

Figure S2. Solid-state ^{13}C NMR spectra of TCT and ACT.
